# CD39 is a negative regulator of P2X7-mediated inflammatory cell death in mast cells

**DOI:** 10.1186/s12964-014-0040-3

**Published:** 2014-07-16

**Authors:** Marcel Kuhny, Thomas Hochdörfer, Cemil Korcan Ayata, Marco Idzko, Michael Huber

**Affiliations:** 1 Institute of Biochemistry and Molecular Immunology, University Clinic, RWTH Aachen University, Pauwelsstr. 30, Aachen, 52074, Germany; 2 Department of Pneumology, University Medical Center, Killianstraße 5, Freiburg, 79106, Germany

**Keywords:** ATP, BMMC, CD39, Cell death, IL-1β, P2X7

## Abstract

**Background:**

Mast cells (MCs) are major contributors to an inflammatory milieu. One of the most potent drivers of inflammation is the cytokine IL-1β, which is produced in the cytoplasm in response to danger signals like LPS. Several controlling mechanisms have been reported which limit the release of IL-1β. Central to this regulation is the NLRP3 inflammasome, activation of which requires a second danger signal with the capacity to subvert the homeostasis of lysosomes and mitochondria. High concentrations of extracellular ATP have the capability to perturb the plasma membrane by activation of P2X7 channels and serve as such a danger signal. In this study we investigate the role of P2X7 channels and the ecto-5´-nucleotidase CD39 in ATP-triggered release of IL-1β from LPS-treated mast cells.

**Results:**

We report that in MCs CD39 sets an activation threshold for the P2X7-dependent inflammatory cell death and concomitant IL-1β release. Knock-out of CD39 or stimulation with non-hydrolysable ATP led to a lower activation threshold for P2X7-dependent responses. We found that stimulation of LPS-primed MCs with high doses of ATP readily induced inflammatory cell death. Yet, cell death-dependent release of IL-1β yielded only minute amounts of IL-1β. Intriguingly, stimulation with low ATP concentrations augmented the production of IL-1β in LPS-primed MCs in a P2X7-independent but caspase-1-dependent manner.

**Conclusion:**

Our study demonstrates that the fine-tuned interplay between ATP and different surface molecules recognizing or modifying ATP can control inflammatory and cell death decisions.

## Background

The innate immune system is essential for the elimination of microbial invaders. It is engaged via germ-line-encoded pattern recognition receptors (PRRs) that recognize diverse pathogen-associated molecular patterns (PAMPs) [[1]] as well as endogenous, danger-associated molecular patterns (DAMPs) [[2],[3]] released during infection or cellular damage [[2],[4]]. Engaged PRRs activate cellular defense mechanisms, which eliminate the imminent threat. Prominent among these mechanisms is the release of the pro-inflammatory cytokine IL-1β. One of the major signaling-hubs of these defense mechanisms is the inflammasome, a multi-protein complex that drives activation of caspase-1. In turn, caspase-1 cleaves pro-IL-1β, an important step preceding the release of the signaling-competent mature IL-1β (mat-IL-1β) [[5]]. A two-step mechanism that requires two distinct signals leads to the activation of the inflammasome. The first signal derives from activation of PRRs, stimulating the NF-κB-dependent transcription of mediators like pro-IL-1β and of components of the inflammasome e.g. the cytoplasmic sensor NACHT, LRR and PYD domains-containing protein 3 (NLRP3). The second signal then activates the NLRP3 inflammasome through a variety of ligands including ATP, crystalline or particular compounds, and bacteria-derived ionophores [[6]]. All of these second stimuli share the ability to subvert the homeostasis of the cell by destabilization of lysosomes and mitochondria or by perturbation of the plasma membrane [[7],[8]]. Thus, pyroptosis, the inflammatory cell death, seems to be an inevitable consequence of inflammasome-engaging second signals.

Among the purinergic receptors P2X7 is the known activator of the NLRP3 inflammasome. It has a low affinity for its sole natural ligand ATP and forms homo-multimeric ion channels with low selectivity for Ca^2+^, Na^+^, and K^+^ [[9]]. Furthermore, P2X7 incorporates pannexin-1 resulting in the formation of non-selective pores permeable to molecules up to 900 Da [[10],[11]]. The exact mechanism by which P2X7 triggers the NLRP3 inflammasome is subject to intensive research. Ultimately, stimulation of the P2X7 receptor with high doses of ATP leads to perturbation of the plasma membrane and subsequent cell death [[9]].

Extracellular ATP is cytotoxic to lymphocytes [[12]]. The ecto-nucleoside triphosphate diphosphohydrolase CD39 converts ATP to AMP, thus limiting the concentrations of extracellular ATP. CD39 has been attributed a protective role in P2X7-mediated apoptosis of endothelial cells [[13]] and a negative regulatory role for mat-IL-1β release from macrophages (MΦ) [[14]]. Accordingly, loss of CD39 promotes lung inflammation upon LPS challenge [[15]].

Released mat-IL-1β mediates a variety of local and systemic responses to infection e.g., induction of fever and promotion of T cell responses [[16]]. These traits confer such impact on inflammatory processes that tight control mechanisms of IL-1β production and release have evolved to protect the host. In fact, diseases classified as auto-inflammatory stem from deregulated IL-1β release [[17]].

Mast cells (MCs) are most recognized for their effector role in the immune response against parasites [[18],[19]]. They line the tissues forming the interface to the outside environment, namely skin, lung and gastro-intestinal tract. Equipped with an array of receptors, MCs sense diverse PAMPs [[1]] and DAMPs [[2],[3]]. Once activated, MCs start the biosynthesis and release of pro-inflammatory mediators e.g., IL-1β and IL-6, as well as immune-regulatory mediators [[1]].

In the present study we demonstrate that CD39 negatively regulates the P2X7-dependent release of IL-1β from LPS-primed bone marrow-derived MCs (BMMCs). In contrast to findings in MΦs and dendritic cells, IL-1β release from BMMCs was causally linked to cell death and did not require processing into mat-IL-1β.

## Results

### Correlation of IL-1β release and cell death in MCs

**Figure 1:**
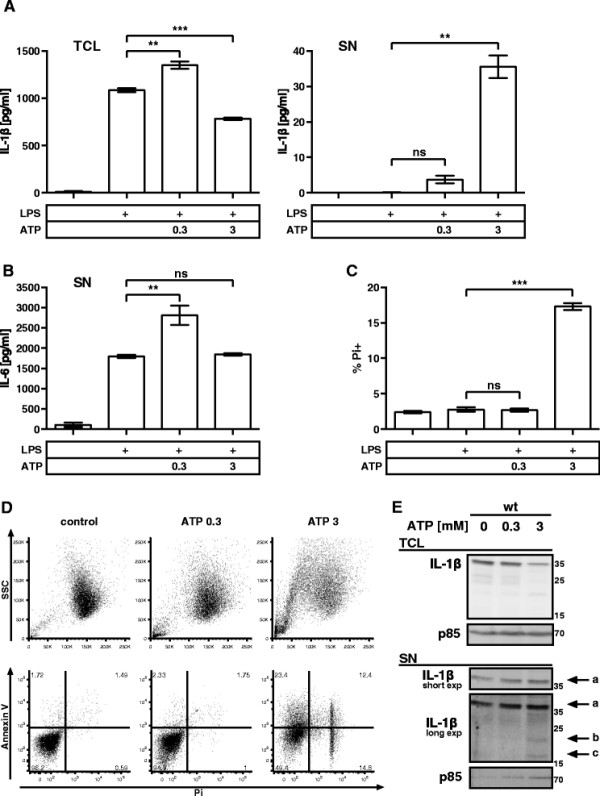
**Correlation of IL-1β release and cell death in mast cells. (A)** wt BMMCs were primed with 1 μg/ml LPS for 3.5 h and then left untreated or stimulated with the indicated concentrations [mM] of ATP for 1 h. TCL and SN were probed for IL-1β by ELISA (n = 8). **(B)** Treatment as in (A); SN was probed for IL-6 by ELISA (n = 4). **(C)** Treatment as in (A); wt BMMCs were stained with Pi and analyzed by FACS (n = 13). **(D)** Treatment as in (A); cells were stained with FITC-conjugated Annexin V and Pi and analyzed by FACS. The morphology is displayed in the forward and side scatter (FSC/SSC); representative result (n = 12). **(E)** wt BMMCs were primed with 1 μg/ml LPS for 3.5 h. The cells were then concentrated to 2*10^6^ cells/60 μL and left untreated or stimulated with the indicated concentrations of ATP for 1 h. TCL and SN were then analyzed by immunoblotting with anti-IL-1β (top and middle panel) and anti-p85 (bottom panel, loading control). For details on indicated bands see text. Shown are means and SD of replicates of one representative experiment each. Statistical analysis of n independent experiments by LMM; FDR-corrected p-values: * < 0.05, ** < 0.005, and *** < 0.0005.

We have previously shown that challenging BMMCs with the TLR4 ligand, LPS, or the endogenous alarmin IL-33 resulted in rapid production and release of IL-6 and TNF-α [[20]]. In parallel, the IL-1β gene was transcribed and pro-IL-1β was produced and retained intracellularly (Additional file 1A-C). In accordance with findings in MΦs, IL-1β processing and subsequent release required a second danger signal. Therefore, we employed the widely used model of ATP stimulation. Thus, excessive amounts of ATP (3 mM), as may be present in areas of tissue injury [[21]], or as consequence of active secretion [[22],[23]], led to the release of IL-1β from BMMCs while the amount of intracellular IL-1β was reduced (Figure 1A). Of note, the used ELISA did not discriminate between pro- and mat-IL-1β. We observed a substantial discrepancy between the amounts of released, and intracellularly retained IL-1β indicating a degradation process during its release. Upon release IL-1β was not further degraded within the 1 h timeframe of ATP stimulation, as the amount of IL-1β in the SN increased time-dependently (Additional file 1D). In contrast to the high dose ATP stimulation (3 mM), addition of low ATP concentrations (0.3 mM) led to an augmented production of pro-IL-1β and IL-6 compared to LPS-primed control cells (Figure 1A + B). Since addition of 3 mM ATP might cause stress to the cells by osmotic strain, we sought to thoroughly assess the viability of the BMMCs after stimulation with ATP by flow cytometry. We found a striking correlation between the release of IL-1β (Figure 1A) and the occurrence of cell death as indicated by propidium iodide (Pi) positive cells (Figure 1C). Furthermore, stimulation with ATP induced dramatic morphological changes in respect to size (FSC) and light refraction (SSC) of the BMMCs (Figure 1D). While 0.3 mM ATP led to a minor increase of the FSC, stimulation with 3 mM ATP induced the formation of a 2nd population with increased SSC and smaller cell bodies. This 2nd population comprised the Pi + cells (Additional file 1F) and increased with the duration of ATP stimulation (Additional file 1E).

Stimulation with 3 mM ATP also resulted in an increase of annexin V (AV) + cells (Figure 1D lower panel). Early exposure of phosphatidylserine (PS) and loss of membrane integrity are characteristics of caspase-1-dependent pyroptosis [[24],[25]]. Accordingly, we expected to find mat-IL-1β rather than pro-IL-1β in the SN of BMMCs stimulated with 3 mM ATP (Figure 1E). To our surprise, we detected predominantly pro-IL-1β (a) but only minute amounts of mat-IL-1β (c). A third band (b) was also present in the SN representing the p20 form of IL-1β, truncated independently of caspase-1 [[26]]. At the same time we found enriched p85, the regulatory subunit of the cytosolic signaling protein PI3K, in the SN when BMMCs were stimulated with 3 mM ATP. We interpreted extracellular p85 as a clear indicator for the breakdown of cellular integrity. These data indicated that pro- and mat-IL-1β might be released from BMMCs in a lytic, cell death-dependent process.

### Caspase-1 controls production of pro-IL-1β

**Figure 2:**
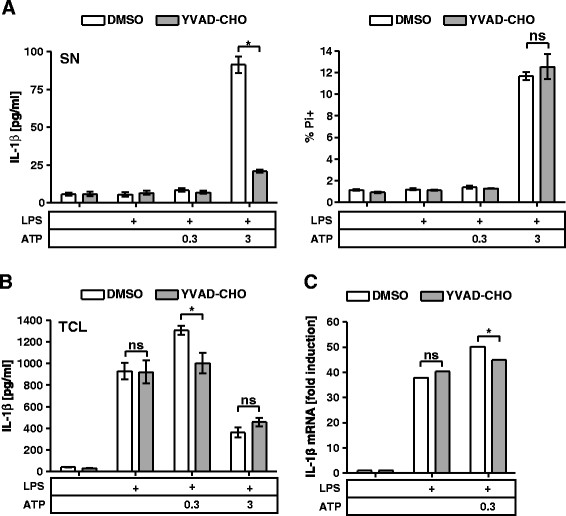
**Caspase-1 controls production of IL-1β in mast cells. (A)** wt BMMCs were primed with 1 μg/ml LPS for 3.5 h and then left untreated or stimulated with the indicated concentrations [mM] of ATP for 1 h; vehicle (DMSO) or caspase-1 inhibitor (YVAD-CHO) was added 1 h prior to stimulation with ATP. TCL and SN were probed for IL-1β by ELISA (n = 5) (left panel). BMMCs were stained with Pi and analyzed by FACS (n = 3) (right panel). **(B)** Treatment as in (A); TCL of wt BMMCs was probed for IL- β by ELISA (n = 5). **(C)** Treatment as in (A); transcripts were analyzed by qPCR (n = 3). Shown are means and SD of replicates of one representative experiment each. Statistical analysis of n independent experiments by LMM; FDR-corrected p-values: * < 0.05, ** < 0.005, and *** < 0.0005.

Pharmacological inhibition of caspase-1 attenuated the amount of released IL-1β without affecting the number of Pi + cells (Figure 2A). However, we found that the augmented intracellular expression of pro-IL-1β protein in response to 0.3 mM ATP was sensitive to caspase-1 inhibition (Figure 2B). This raised the question whether the observed reduction in IL-1β release in the presence of the caspase-1 inhibitor was actually due to the inability of caspase-1 to facilitate the processing and release of IL-1β or rather the lack of caspase-1-dependent augmented production of pro-IL-1β. By qPCR analysis we found that the augmented production of pro-IL-1β was also evident on transcript level. This indicated that the observed boost in pro-IL-1β originated from enhanced transcription rather than a modulation of the translation. Application of the caspase-1 inhibitor nullified the enhancing effect of 0.3 mM ATP on the transcript level (Figure 2C).

Taken together, these findings suggest that caspase-1 is not required for the ATP-triggered inflammatory cell death in BMMCs. Yet, caspase-1 seems to regulate the amount of released IL-1β, at least in part, by influencing the transcription and biosynthesis of pro-IL-1β.

### P2X7 is required for cell death and IL-1β release

**Figure 3:**
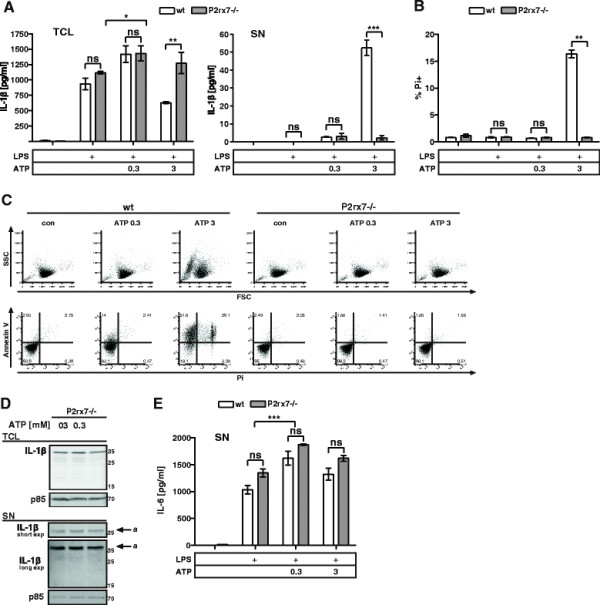
**P2X7 is required for ATP-induced IL-1β release and cell death. (A)** wt and P2rx7−/− BMMCs were primed with 1 μg/ml LPS for 3.5 h and then left untreated or stimulated with the indicated concentrations [mM] of ATP for 1 h. TCL and SN were probed for IL-1β by ELISA (n = 5). **(B)** Treatment as in (A); wt and P2rx7−/− BMMCs were stained with Pi and analyzed by FACS (n = 4). **(C)** Treatment as in (A); cells were stained with FITC-conjugated Annexin V and Pi and analyzed by FACS. The morphology is displayed in the forward and side scatter (FSC/SSC); representative result (n = 4). **(D)** P2rx7−/− BMMCs were primed with 1 μg/ml LPS for 3.5 h. Cells were then concentrated to 2*10^6^ cells/60 μL and left untreated or stimulated with the indicated concentrations of ATP for 1 h. TCL and SN of wt and P2rx7−/− BMMCs were then analyzed by immunoblotting with anti-IL-1β (top and middle panel) and anti p85 (bottom panel, loading control). For details on indicated bands see text. **(E)** Treatment as in (A); SN were probed for IL-6 by ELISA (n = 6). Shown are means and SD of replicates of one representative experiment each. Statistical analysis of n independent experiments by LMM; FDR-corrected p-values: * < 0.05, ** < 0.005, and *** < 0.0005.

BMMCs express transcripts of several ATP-specific receptors of the P2 family (Additional file 2A). Prominently, they express P2X7, a low-affinity receptor for ATP. 300 μM ATP have been demonstrated to induce the formation of multimeric P2X7 ion-channels [[27]]. At concentrations in the mM range, pannexin-1 gets incorporated into P2X7 pores allowing passage of molecules up to 900 Da [[10],[28]]. Because of the correlation between cell death, IL-1β release and the presence of pro- and mat-IL-1β in the SN, we tested the requirement for P2X7 in ATP-induced cell death and IL-1β release. To this end, we generated P2rx7−/− BMMCs from respective knockout mice. After four weeks of culture, differentiation of wt and P2rx7−/− BMMCs was comparable according to expression of FcεRI and Kit (Additional file 2B). As expected, LPS-primed P2rx7−/− BMMCs treated with 3 mM ATP did not release IL-1β (Figure 3A). In line with our previous results, P2rx7−/− cells showed no cell death upon ATP stimulation either (Figure 3B). In fact, P2rx7−/− BMMCs were completely protected from the detrimental effects of ATP stimulation. The hallmark 2nd population in samples of wt BMMCs treated with 3 mM ATP was absent in cultures of ATP-treated P2rx7−/− cells as well as the above mentioned changes in the FSC and SSC (Figure 3C). Another striking difference was the lack of PS on the surface of P2rx7−/− BMMCs in contrast to wt cells treated with 3 mM ATP (Figure 3C lower panel). We concluded that the initiation of ATP-induced inflammatory cell death of BMMCs strictly required the action of P2X7. We further tested for processing and release of IL-1β (Figure 3D); yet, we could not detect mat-IL-1β in the SN and pro-IL-1β (a) did not rise above background levels with ATP stimulation. Noteworthy, increased production of pro-IL-1β (as measured in the TCL) and IL-6 induced by 0.3 mM ATP were independent of P2X7 (Figure 3A + E). Consequently, BMMC stimulation with ATP must induce P2X7-independent signaling pathways as well. We observed transient activation of the MAPKs ERK1/2 in wt cells from 1 to 5 min after stimulation with 0.3 mM ATP. In contrast, P2rx7−/− BMMCs showed an even shorter phosphorylation of ERK1/2 (Additional file 3A upper panel). We also found that stimulation with 0.3 mM ATP induced a rapid rise in intracellular Ca^2+^ levels, which slowly declined over time. This Ca^2+^ flux was sensitive to the P2X7 antagonist KN-62 save for an initial Ca^2+^ peak (Additional file 3B). The same initial spike in Ca^2+^ levels could be observed in P2rx7−/− BMMCs stimulated with 0.3 mM ATP. This spike was insensitive to KN-62, in line with a contribution of other ATP-sensitive receptors than P2X7. However, activation of ERK1/2 and Ca^2+^ mobilization served merely as readouts for P2X7-independent signaling and their consequences with respect to the observed augmented cytokine production remain elusive as interference with ERK signaling or Ca^2+^ mobilization could not significantly alter cytokine production (not shown).

So far, these data suggest that ATP-triggered cell death and release of IL-1β are initiated in a P2X7-dependent manner (Figure 3C, D, and E); yet, ATP initiates signaling (Additional file 3A + B) and affects production of pro-IL-1β and IL-6 (Figure 3A + B) in a P2X7-independent manner.

### CD39 is a negative regulator of ATP-induced IL-1β release and cell death

**Figure 4:**
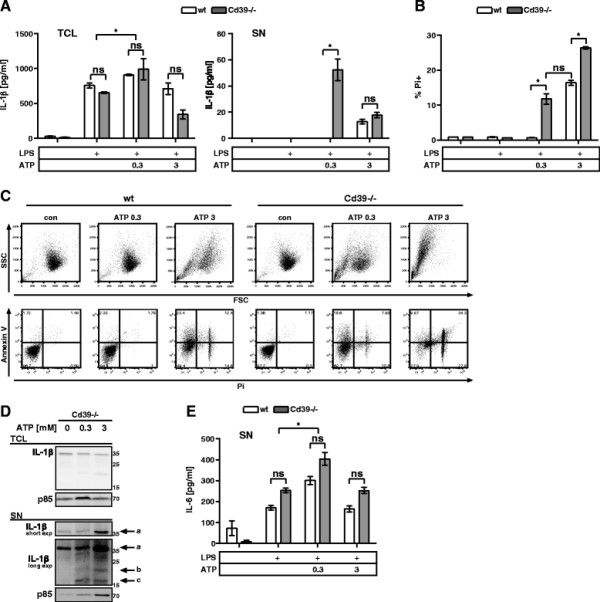
**CD39 is a negative regulator of IL-1β release and cell death. (A)** wt and Cd39−/− BMMCs were primed with 1 μg/ml LPS for 3.5 h and then left untreated or stimulated with the indicated concentrations [mM] of ATP for 1 h. TCL and SN were probed for IL-1β by ELISA (n = 5). **(B)** Treatment as in (A); wt and Cd39−/− BMMCs were stained with Pi and analyzed by FACS (n = 6). **(C)** Treatment as in (A); cells were stained with FITC-conjugated Annexin V and Pi and analyzed by FACS. The morphology is displayed in the forward and side scatter (FSC/SSC); representative result (n = 4). **(D)** Cd39−/− BMMCs were primed with 1 μg/ml LPS for 3.5 h. The cells were then concentrated to 2*10^6^ cells/60 μL and left untreated or stimulated with the indicated concentrations of ATP for 1 h. TCL and SN were then analyzed by immunoblotting with anti-IL-1β (top and middle panel) and anti-p85 (bottom panel, loading control). For details on indicated bands see text. **(E)** Treatment as in (A); SN of wt and Cd39−/− BMMCs were probed for IL-6 by ELISA (n = 6). Shown are means and SD of replicates of one representative experiment each. Statistical analysis of n independent experiments by LMM; FDR-corrected p-values: * < 0.05, ** < 0.005, and *** < 0.0005.

Since the release of IL-1β and the concomitant cell death required high concentrations of ATP, ecto-nucleotidases on the MC surface could set the threshold for ATP-induced cell death and IL-1β release. The ecto-nucleotidase CD39 has been implicated in the regulation of ATP-induced responses of MΦs and is also expressed on MCs (Additional file 2A). We generated BMMCs from Cd39−/− mice, which developed comparably to wt cells (Additional file 2B). Strikingly, these BMMCs released IL-1β even at 0.3 mM ATP as efficiently as wt BMMCs stimulated with 3 mM ATP (Figure 4A). In agreement with our previous findings, Cd39−/− BMMCs were highly susceptible to ATP-induced cell death, indicated by a significant Pi + cell population already after stimulation with 0.3 mM ATP (Figure 4B). Furthermore, Cd39−/− BMMCs stimulated with 0.3 mM ATP showed a comparable AV/Pi staining pattern as wt BMMCs stimulated with 3 mM ATP. Under these conditions the morphological changes (FSC, SSC) of Cd39−/− cells were comparable to those observed in wt BMMCs at 10 fold higher concentrations of ATP (Figure 4C). Along this line, the destructive impact of 3 mM ATP on Cd39−/− BMMCs was much stronger when compared to wt cells. Since this higher susceptibility of Cd39−/− BMMCs might also encompass a higher efficiency in IL-1β processing, we tested for processing and release of pro-IL-1β (Figure 4D). Indeed, analysis of concentrated SN from ATP-stimulated Cd39−/− BMMCs revealed a faint band of about 17 kDa (c) when the cells were stimulated with 0.3 mM ATP and, slightly stronger, with 3 mM ATP. As described earlier, the p20 form of IL-1β (b) was also detectable in the SN. The LPS-induced production of IL-6 seemed to be unaffected by the loss of CD39 (Figure 4E). Augmented pro-IL-1β and IL-6 production upon low dose ATP stimulation were unaltered in Cd39−/− BMMCs. These data led us to hypothesize that the absence of CD39 led to a sustained stimulation with high concentrations of ATP, which accounted for the increased susceptibility of Cd39−/− BMMCs to ATP-induced inflammatory cell death and IL-1β release.

### Non-hydrolysable ATP aggravates cell death and IL-1β release

**Figure 5:**
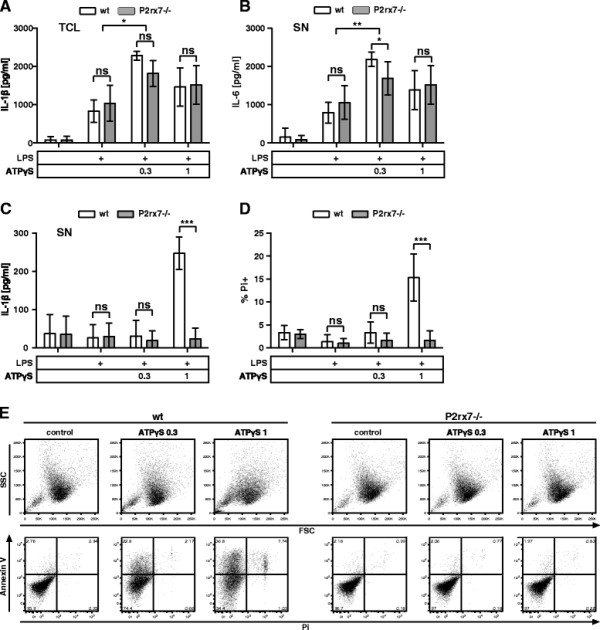
**Stimulation with ATPγS aggravates cell death and IL-1β release. (A)** wt and P2rx7−/− BMMCs were primed with 1 μg/ml LPS for 3.5 h and then left untreated or stimulated with the indicated concentrations [mM] of ATPγS for 1 h. TCL was probed for IL-1β by ELISA (n = 4). **(B)** Treatment as in (A); SN of wt and P2rx7−/− BMMCs were probed for IL-6 by ELISA (n = 4). **(C)** Treatment as in (A); SN of wt and P2rx7−/− BMMCs were probed for IL-1β by ELISA (n = 4). **(D)** Treatment as in (A); wt and P2rx7−/− BMMCs were stained with Pi and analyzed by FACS (n = 3). **(E)** Treatment as in (A); cells were stained with FITC-conjugated Annexin V and Pi and analyzed by FACS. The morphology is displayed in the forward and side scatter (FSC/SSC); representative result (n = 3). Shown are means and SD of replicates of one representative experiment each. Statistical analysis of n independent experiments by LMM; FDR-corrected p-values: * < 0.05, ** < 0.005, and *** < 0.0005.

Extracellular ATP has a short half-life and is efficiently degraded by CD39 [[29]]. Consequently, the use of ATPγS, a non-hydrolysable derivate of ATP, should mimic the phenotype of Cd39−/− BMMCs and exert its effects at considerably lower concentrations than ATP. Stimulation of LPS-primed wt BMMCs with up to 0.3 mM ATPγS increased the production of IL-1β and IL-6 (Figure 5A + B) while stimulations with 1 mM ATPγS induced cell death and release of IL-1β (Figure 5C + D). 1 mM ATPγS also induced the morphological changes and the AV/Pi staining pattern of cell death as observed in wt BMMCs stimulated with 3 mM ATP (Figure 5E). As predicted, ATPγS was more efficient than ATP, with effective concentrations about 3 fold lower than the amounts of ATP. In support of the idea that persistent stimulation by ATPγS might be accountable for its increased efficiency over ATP, we observed a prolonged activation of ERK1/2 when wt BMMCs were stimulated with ATPγS rather than ATP (Additional file 3A). In agreement with the pivotal role of P2X7, ATPγS-mediated cell death and IL-1β release (Figure 5A-E), as well as prolonged signaling (Additional file 3A lower panel) were completely absent in P2rx7−/− BMMCs.

## Discussion

In this study we report that CD39 is a negative regulator of ATP-induced inflammatory cell death and IL-1β release in MCs. The loss of CD39 significantly lowered the threshold for ATP-triggered IL-1β release and cell death. We corroborated these findings by using the non-hydrolysable ATPγS, which mimicked the Cd39−/− phenotype. Our results confirm and extend the pivotal role of the ATP receptor P2X7 in initiating IL-1β release and cell death. We also found ATP-induced P2X7-independent signaling, which substantially contributed to the production of pro-IL-1β and IL-6. Lastly, the ATP-induced release of IL-1β from MCs seems to be partially independent of caspase-1 activity since pro-IL-1β was found in higher abundance than mat-IL-1β in the SN of ATP-stimulated MCs.

CD39 plays a pivotal role in the regulation of the physiological concentrations of extracellular nucleotides in the mucosa [[30]]. Specifically for MΦs, CD39 has been shown to modulate the P2X7-dependent activation of the NLRP3 inflammasome [[14]]. On a broader scale, Théâtre et al. recently showed that in transgenic mice overexpressing CD39 exceedingly reduced concentrations of extracellular ATP promoted lung inflammation in response to LPS administration [[31]]. Upon challenge they observed enhanced recruitment of neutrophils and MΦs, elevated levels of IL-6 and other inflammatory factors but not IL-1β in the bronchoalveolar lavage (BAL). One could hypothesize that the decreased ATP concentrations in CD39 overexpressing mice resulted in sub-lethal stimulation of immune cells. This in turn led to augmented cytokine levels, except IL-1β, in the BAL. Stimulation of LPS-primed wt BMMCs with low doses of ATP nicely reflected and partly explained these findings. These cells secreted substantially more IL-6 and produced more pro-IL-1β compared to BMMCs primed with LPS alone, while secretion of IL-1β was unaltered. In line with these findings stimulation of Cd39−/− BMMCs with low doses of ATP already induced release of IL-1β and morphological signs of cell death comparable to wt BMMCs stimulated with 10 fold more ATP. As an alternative approach we employed ATPγS, which led to IL-1β release and cell death at 3 fold lower concentrations than ATP, in a completely P2X7-dependent manner. Considering the lower potency of ATPγS to activate P2X7 compared to ATP [[32]], these results mimic the Cd39−/− phenotype and thus support the negative role for CD39 on P2X7-mediated responses in BMMCs. The efficient removal of extracellular ATP by CD39 together with the low affinity of P2X7 for ATP [[27]] represent a stringent activation threshold preventing premature IL-1β release from MCs at sites of inflammation or tissue damage. However, this threshold could be biased according to the type of tissue and/or the nature of the thread by modulation of CD39 expression.

In an attempt to comprehensively study the release of IL-1β from BMMCs we also monitored the production of intracellular pro-IL-1β and its expected reduction during ATP-induced release. We observed augmented pro-IL-1β production in LPS-primed BMMCs after stimulation with low doses of ATP. The same applied for the production of IL-6. This effect was not dependent on P2X7, yet originated from ATP-induced signaling since stimulation of Cd39−/− BMMCs with ATP or ATPγS treatment of wt cells, both of which forestalled the conversion of ATP to AMP and adenosine, yielded comparable results. We observed P2X7-independent mobilization of cellular Ca^2+^ flux and activation of the MAPKs ERK1/2 after stimulation with low doses of ATP (Additional file 3A + B). The very short-lived phosphorylation of ERK1/2 and the equally brief spike in Ca^2+^ mobilization resembled the kinetic footprint of G-protein coupled receptors (GPCR). In all likelihood, these GPCRs are members of the P2Y subfamily of ATP receptors (Additional file 2A), which have also been implicated in the degranulation of MCs [[33]].

Our observations regarding the release of pro-IL-1β and the concomitant cell death after stimulation with high doses of ATP are in contrast to the established model of IL-1β secretion during pyroptosis, which is characterized by the cleavage of pro-IL-1β into the mature and signaling competent form [[34],[35]]. Western blot analysis revealed that LPS-primed BMMCs predominantly released pro-IL-1β when stimulated with high doses of ATP. Two minor bands were also present: the p20 form of IL-1β [[26]] and mat-IL-1β at 17 kDa. We further observed that stimulation with ATP induced the release of β-hexosaminidase, a common readout for degranulation (not shown). It is thus likely that upon stimulation of BMMCs with high doses of ATP, released pro-IL-1β encountered active MC-derived proteases in the extracellular space. This could result in caspase-1-independent processing of pro-IL-1β as has been reported for MC- and neutrophil-derived chymases, elastases [[36],[37]], and others [[38]-[40]]. This process might be aggravated in the context of allergic diseases where IgE-triggered degranulation might enhance extracellular conversion of pro- into mat-IL-1β. However, co-stimulation of LPS-primed BMMCs with antigen resulted in attenuated production of pro-IL-1β [[20]] which would imply a generally attenuated production of pro-IL-1β. Hence, pro-IL-1β could be converted into mat-IL-1β in a caspase-1-independent manner after its release from MCs.

We also observed a striking discrepancy between the decrease of pro-IL-1β in the TCL and the minute amounts of IL-1β actually detectable in the SN by ELISA. A partial degradation of IL-1β by endolysosomal processes in monocytes [[41]] and the involvement of autophagosomal degradation has been reported [[42]]. MCs are packed with protease-filled secretory granules. Thus, upon disruption of the membrane homeostasis and lysosome destabilization by excessive amounts of extracellular ATP, the contents of these granules could effectively cleave and ultimately inactivate a large proportion of IL-1β. Though, preliminary data indicate that inhibition of chymotrypsin and trypsin-like proteases rescues a certain amount of extracellular IL-1β (not shown), the plethora of MC-derived proteases in the SN makes it extremely difficult to discern productive cleavage of pro-IL-1β into mat-IL-1β from degradation.

Several unconventional release modes have been proposed for IL-1β depending on the cell type and nature of the stimuli [[5]]. In this work, BMMCs treated with high doses of ATP displayed a strict correlation between IL-1β release and cell death suggesting a terminal release mechanism similar to pyroptosis. Indeed, high doses of ATP induced early exposure of PS on the outer leaflet of the plasma membrane (PM) and the hallmark release of IL-1β. Pyroptosis is driven by active caspase-1, which facilitates the processing and release of IL-1β and the onset of programmed cell death [[43],[44]]. Indeed, inhibition of caspase-1 reduced the release of IL-1β after stimulation with high doses of ATP but it also significantly reduced the augmented pro-IL-1β production of LPS-primed BMMCs after stimulation with low doses of ATP (Figure 2A-C). This finding is in line with reports that caspase-1 modulates the activity of NF-κB-dependent gene expression [[45],[46]] and warrants caution when interpreting the impact of caspase-1 inhibition based solely on the release of IL-1β. Furthermore, inhibition of caspase-1 failed to reduce ATP-triggered cell death. Recent work has implicated non-canonical and caspase-1-independent functions of the inflammasome. Via yet unknown receptors, the inflammasome senses the presence of cytosolic LPS and leads to the release of IL-1β and to pyroptosis in a caspase-11-dependent manner [[47]]. The challenge of future research will be to separate the canonical from the non-canonical activation of the inflammasome after stimulation with any danger signal that perturbs the membrane integrity. In summary, stimulation with high doses of ATP induced P2X7-dependent but caspase-1-independent inflammatory cell death, which might be categorized as non-canonical pyroptosis.

## Conclusions

Since MCs are located in diverse tissues, a site-specific immune response must be mounted to fight pathogens while avoiding undue inflammation and tissue damage. Therefore, MC subtypes are shaped by the microenvironment within the diverse tissues. This ensures that MCs provide a tailored immune response by releasing a tissue-specific profile of bioactive mediators [[48]]. Differential control of the ATP-induced release of IL-1β could be a consequence of these site-specific MC subtypes also reflected in different results gained by varying BMMC culture techniques [[49]]. CD39 appears to be a good candidate to further investigate the differential regulation between MC subtypes. It is not only able to set a threshold for ATP-induced activation of P2X7 but further provides new ligands for other purinergic receptors by processing ATP, in combination with CD73, to adenosine.

## Methods

### Cell culture

According to procedures established by Razin et al. [[50]], bone marrow cells (2×10^6^/ml) from 6 to 8 week old male mice (129/Sv × C57Bl/6) were cultured (37°C, 5% CO_2_) as single cell suspensions in culture medium (RPMI 1640 medium containing 12% FCS, 1% X63Ag8-653-conditioned medium, as a source of IL-3 [[51]], 2 mM L-glutamine, 1×10^-5^ M 2-mercaptoethanol, 50 units/ml penicillin, and 50 mg/ml streptomycin). At weekly intervals, the non-adherent cells were reseeded at 1×10^6^ cells/ml in fresh medium. By 4–5 weeks in culture, greater than 99% of the cells were kit and FcεR1 positive as assessed by phycoerythrin-labeled anti-kit antibodies (Pharmingen, Mississauga, Canada) and FITC-labeled rat anti-mouse IgE antibodies (Southern Biotechnology, Birmingham, AL, USA), respectively. P2rx7−/− and Cd39−/− BMMCs were *in vitro* differentiated using the same protocol but starting from bone marrow cells of 6 to 8 week old P2rx7−/− and Cd39−/− (both C57Bl/6) mice.

### Reagents

R-form LPS from S. minnesota mutant R595 was extracted and purified as described [[52],[53]] and was a gift from M. Freudenberg and C. Galanos (MPI for Immunobiology, Freiburg, Germany). The synthetic lipopetide FSL-1 was obtained from Echaz Microcollections (Tübingen, Germany). IL-33 was purchased from Axxora Deutschland GmbH (Grünberg, Germany). ATP and ATPγS were purchased from Sigma (Germany). DMSO was purchased from Carl Roth GmbH & Co (Karlsruhe, Germany).

### Stimulation of mast cells

BMMCs were supplied with fresh culture medium over night to ensure maximum viability. The cells were resuspended in stimulation medium (growth medium w/o IL-3) at a density of 1×10^6^/ml and transferred to 96well plates. Upon stimulation as indicated in the figure legends, the SN and TCL were separated by centrifugation and further analyzed.

### Cytokine ELISA

Mouse IL-6 ELISAs and mouse IL-1β ELISAs (BD, Heidelberg, Germany) were performed according to the manufacturer’s instructions. IL-6 was measured in supernatants. IL-1β was measured from total cell lysates (TCL) and supernatants (SN). Levels of cytokines varied between experiments due to genetic background or age of the cells. Qualitative differences or similarities between WT and mutant cells, however, were consistent throughout the study.

### Flow cytometry

BMMCs were stained with FITC-conjugated Annexin V (ImmunoTools, Friesoythe, Germany) and Pi (Sigma, Germany) for 15 min and analyzed by flow cytometry using a FACScanto II (BD, Heidelberg, Germany). The settings of the flow cytometer were identical for all measurements within each experiment. The acquired data were further analyzed using FlowJo analysis software (Tree Star, Ashland, USA). Unless stated otherwise, the figures represent ungated, total events.

### Western blotting

BMMCs were pelleted and solubilized with 0.5% NP-40 and 0.1% Na-deoxycholate in phosphorylation solubilization buffer at 4°C [[54]]. The postnuclear supernatants were subjected directly to SDS-PAGE and Western blot analysis as described previously [[55]]. Anti-P-ERK 1/2 was purchased from Cell Signaling Technologies (Danvers, USA), anti-p85 from Millipore (Billerica, USA), anti-actin from Santa Cruz Biotechnology (Dallas, USA), and anti-IL-1β from R&D Systems (Minneapolis, USA).

### RT-qPCR

Total RNA of 4*10^6^ cells was extracted using the RNeasy Mini Kit (Qiagen) according to the manufacturer's instructions. RNA (1 μg) was reverse transcribed using Random hexamers (Roche) and Omniscript RT Kit (Qiagen) according to the manufacturer's instructions. qPCR was performed on a Rotorgene (Qiagen) with Sybr green reaction mix (Bioline #QT650-02). Expression of IL-1β transcript was normalized to the housekeeper mGUSB (Qiagen). Primer: IL-1β fwd; AAC CTG CTG GTG TGT GAC GTT C, rev; CAG CAC GAG GCT TTT TTG TTG T; eff.: 0.99029, Gusb (Qiagan) cat. # QT00176715; eff.: 1.01478.

### Statistical analysis

Data generated from independent experiments were analyzed by a linear mixed model (LMM) using the least square mean differences approach followed by unpaired, two-tailed t-test. Resulting p-values were adjusted for multiple comparisons by false discovery rate (FDR). Figures represent means and SD of replicates of one representative experiment each. Statistical analysis of n independent experiments (with n indicated in the respective figure legends). p-values of * < 0.05, ** < 0.005, and *** < 0.0005 were considered statistically significant. All statistical analysis was performed using JMP ver. 10 (SAS, Cary NC, USA).

## Abbreviations

AMP: Adenosine monophosphat

ATP: Adenosine 5’-triphosphate

ATPγS: Adenosine 5’-[γ-thio]triphosphate

AV: Annexin V

BAL: Bronchoalveolar lavage

BMMC: Bone marrow-derived mast cell

DAMP: Damage-associated molecular pattern

DC: Dendritic cell

ERK1/2: Extracellular signal-regulated kinase1/2

FACS: Fluorescence-activated cell sorting

FSC: Forward scatter

IL-1β: Interleukin-1β

MAPK: Mitogen-activated protein kinase

MT: Mitochondria

MΦ: Macrophage

NLRP3: NOD-like receptor family, pyrin domain containing 3

PAMP: Pathogen-associated molecular pattern

Pi: Propidium iodide

PRR: Pattern recognition receptor

PS: Phosphadidyl serine

SN: Supernatant

SSC: Side scatter

TCL: Total cell lysate

TLR: Toll-like receptor

TNF-α: Tumor necrosis factor-α

## Competing interests

The authors declare that they have no competing interests.

## Authors’ contributions

MK has performed most of the experiments, interpreted the data and wrote the manuscript. TH and CKA performed experiments. MI contributed indispensable reagents. MH has initiated and designed the study, interpreted the data and critically revised the manuscript. All authors have read and approved the final manuscript.

## Additional files

## Supplementary Material

Additional file 1: **Production and release of IL-1β are coupled to cell fate.** A) wt BMMCs were primed with 1 μg/ml LPS or 2 ng/ml IL-33 for 3.5 h. TCL and SN were probed for IL-1β by ELISA (n=3). B) 4*10^6^ wt BMMCs were treated as in A. qPCR was performed using primers for IL-1β normalized to Gusb (n=3). C) wt BMMCs were left untreated or primed with 2 ng/ml IL-33 or 1 μg/ml LPS for 3.5 h. TCL was analyzed by immunoblotting with anti-IL-1β and anti-actin antibodies (loading control). The equivalent of 3*10^5^ has been loaded per lane. D) wt BMMCs were primed with 1 μg/ml LPS for 3.5 h and then left untreated or stimulated with 3 mM ATP for the indicated times. SN was probed for IL-1β by ELISA (n=3). E) wt BMMCs were treated as in D, stained with Pi and analyzed by FACS (n=3). F) wt BMMCs were treated as in A, stained with FITC-conjugated Annexin V (AV) and Pi and analyzed by FACS. Gates were positioned to circumference the two distinct populations apparent in the FSC/SSC dot plot and the respective staining patterns for AV/Pi were displayed in the assigned dot plots. Click here for file

Additional file 2: **Expression of P2Rs and CD39 on BMMCs and differentiation control.** A) Quantitative PCR was performed on a LightCyler 480 (Roche, Mannheim, Germany) using the Fast Blue qPCR Mastermix Plus kit that includes UNG (Uracyl-N-glycosylase for carry-over prevention), (Eurogentec, Cologne, Germany). β2-microglobulin (β2m) was used as reference gene, forward primer: 5’_GGTGCTTGTCTCACTGAC_3’, probe: 5’_FAM-ATGCTATGCACAAAACGCCTC-BHQ-1_3’ reverse primer: 5’_GTTCGGCTTCCCATTCTC_3’, efficiency: 99.4%, locked nucleic acids are shown with an underline. Relative quantification analysis and primer/probe design were done as previously described [[56]]. Primers for P2rx1: Mm00435460_m1, P2rx3: Mm00523701_g1, P2rx4: Mm00501795_g1, P2rx7: Mm01199500_m1, P2ry1: Mm02619947_s1, P2ry2: Mm04207602_m1, P2ry4: Mm00445136_s1, P2ry6: Mm01275473_m1, P2ry12: Mm01283320_m1, P2ry13: Mm00546978_m1, P2ry14: Mm01289602_m1, CD39: Mm00515447_m1, and CD73: Mm00501915_m1 from Applied Biosystems (Darmstadt, Germany). B) After 4 weeks in culture wt, P2rx7-/-, and Cd39-/- BMMCs were probed with anti-FcεRI-FITC (eBioscience, San Diego, USA) and anti-Kit-PE (BD, Heidelberg, Germany) antibodies for surface expression of respective receptors. Click here for file

Additional file 3: **P2X7-independent ATP signaling in BMMCs.** A) wt and P2rx7-/- BMMCs were primed with 1 μg/ml LPS for 3.5 h and then left untreated or stimulated with 0.3 mM ATP or 0.3 mM ATPγS for the indicated times. TCL were then subjected to anti-P-Erk1/2 (upper panel) and anti-p85 (lower panel) immunoblotting. B) wt and P2rx7-/- BMMCs were stained with Ca2+-sensitive dyes Fura Red-AM and Fluo-3-AM (invitrogen, Darmstadt, Germany) in RPMI containing 12% FCS for 40 min at 37°C. Ca2+ flux was monitored by flow cytometry. Baseline fluorescence intensities of both dyes were set to the same height. After 30 sec vehicle or KN-62 was added for 5 min and than ATP [0.3 mM] was added for another 5 min. The ratio of Fluo-3/Fura Red * 10 was calculated and the results were converted to kinetics using FlowJo analysis software. Click here for file
