# Supplementary figures and images for: CD39 is a negative regulator of P2X7-mediated inflammatory cell death in mast cells
Source: Cell Commun Signal. 2014 Jul 16;12:40. doi: 10.1186/s12964-014-0040-3 (PMC4110707; doi:10.1186/s12964-014-0040-3)

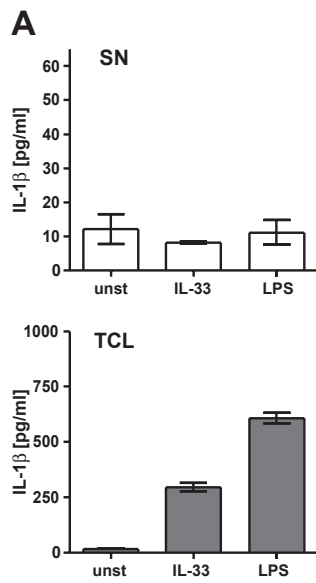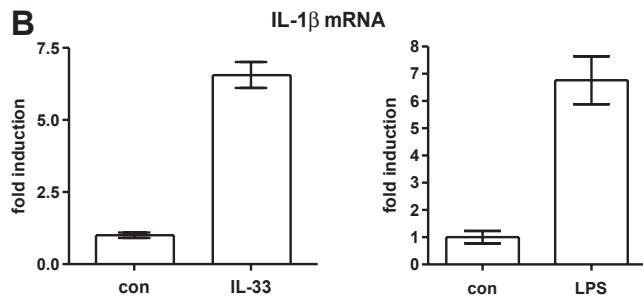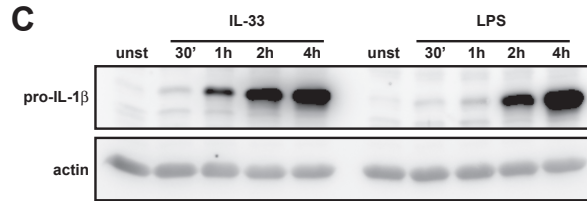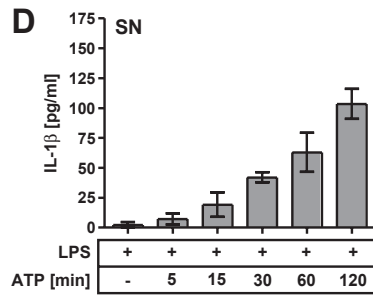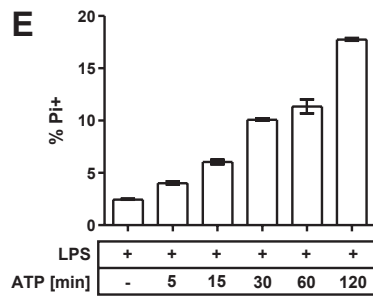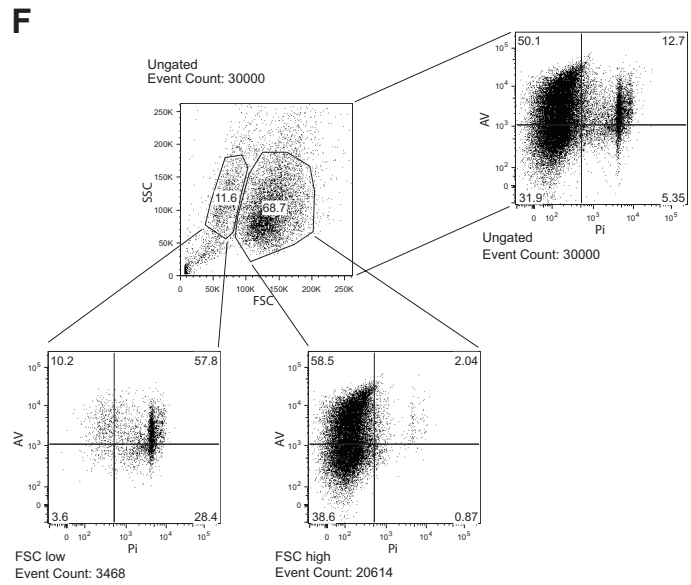

Supplement: Additional file 1: — Production and release of IL-1β are coupled to cell fate. A) wt BMMCs were primed with 1 μg/ml LPS or 2 ng/ml IL-33 for 3.5 h. TCL and SN were probed for IL-1β by ELISA (n=3). B) 4*106 wt BMMCs were treated as in A. qPCR was performed using primers for IL-1β normalized to Gusb (n=3). C) wt BMMCs were left untreated or primed with 2 ng/ml IL-33 or 1 μg/ml LPS for 3.5 h. TCL was analyzed by immunoblotting with anti-IL-1β and anti-actin antibodies (loading control). The equivalent of 3*105 has been loaded per lane. D) wt BMMCs were primed with 1 μg/ml LPS for 3.5 h and then left untreated or stimulated with 3 mM ATP for the indicated times. SN was probed for IL-1β by ELISA (n=3). E) wt BMMCs were treated as in D, stained with Pi and analyzed by FACS (n=3). F) wt BMMCs were treated as in A, stained with FITC-conjugated Annexin V (AV) and Pi and analyzed by FACS. Gates were positioned to circumference the two distinct populations apparent in the FSC/SSC dot plot and the respective staining patterns for AV/Pi were displayed in the assigned dot plots. [file s12964-014-0040-3-S1.pdf]

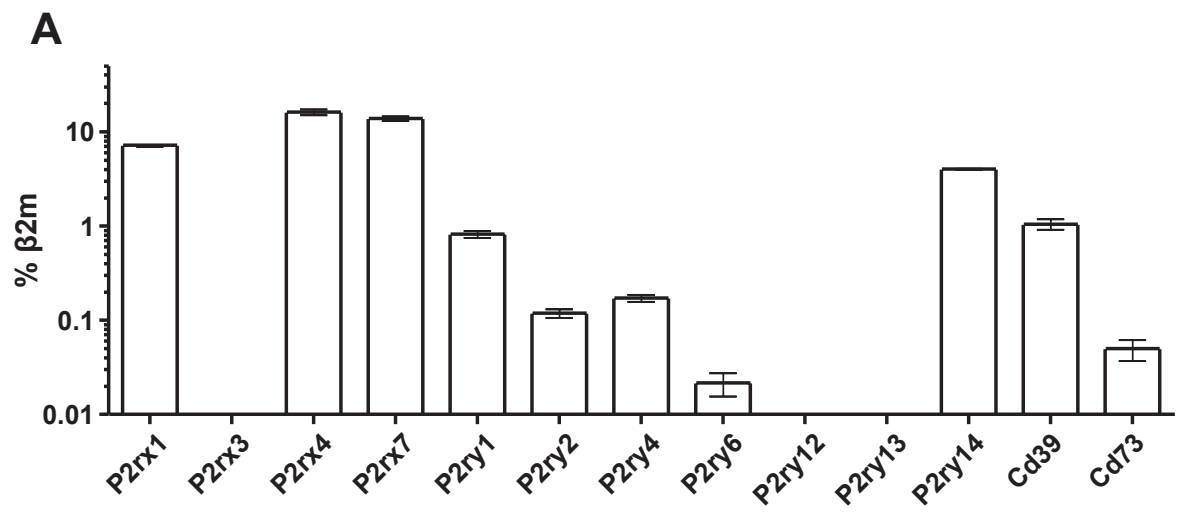

**B**

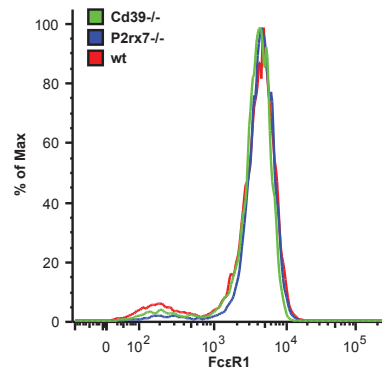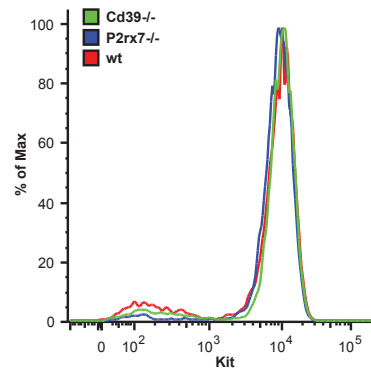

Supplement: Additional file 2: — Expression of P2Rs and CD39 on BMMCs and differentiation control. A) Quantitative PCR was performed on a LightCyler 480 (Roche, Mannheim, Germany) using the Fast Blue qPCR Mastermix Plus kit that includes UNG (Uracyl-N-glycosylase for carry-over prevention), (Eurogentec, Cologne, Germany). β2-microglobulin (β2m) was used as reference gene, forward primer: 5’_GGTGCTTGTCTCACTGAC_3’, probe: 5’_FAM-ATGCTATGCACAAAACGCCTC-BHQ-1_3’ reverse primer: 5’_GTTCGGCTTCCCATTCTC_3’, efficiency: 99.4%, locked nucleic acids are shown with an underline. Relative quantification analysis and primer/probe design were done as previously described [[56]]. Primers for P2rx1: Mm00435460_m1, P2rx3: Mm00523701_g1, P2rx4: Mm00501795_g1, P2rx7: Mm01199500_m1, P2ry1: Mm02619947_s1, P2ry2: Mm04207602_m1, P2ry4: Mm00445136_s1, P2ry6: Mm01275473_m1, P2ry12: Mm01283320_m1, P2ry13: Mm00546978_m1, P2ry14: Mm01289602_m1, CD39: Mm00515447_m1, and CD73: Mm00501915_m1 from Applied Biosystems (Darmstadt, Germany). B) After 4 weeks in culture wt, P2rx7-/-, and Cd39-/- BMMCs were probed with anti-FcεRI-FITC (eBioscience, San Diego, USA) and anti-Kit-PE (BD, Heidelberg, Germany) antibodies for surface expression of respective receptors. [file s12964-014-0040-3-S2.pdf]

**A**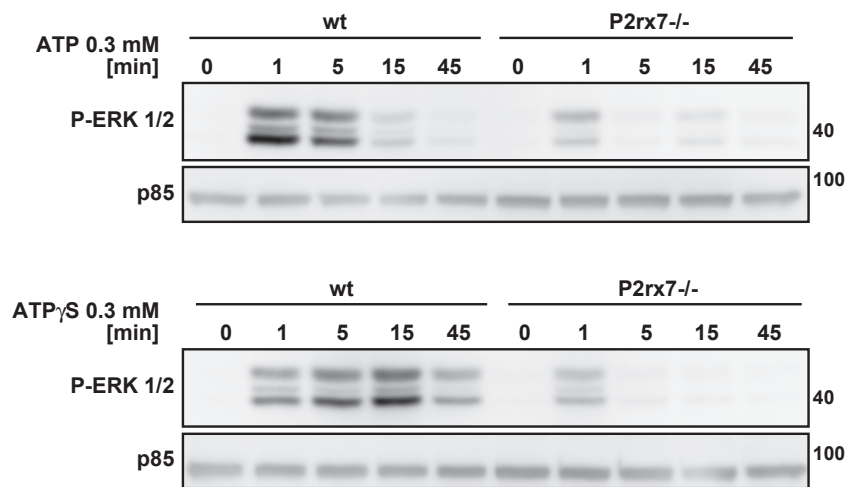**B**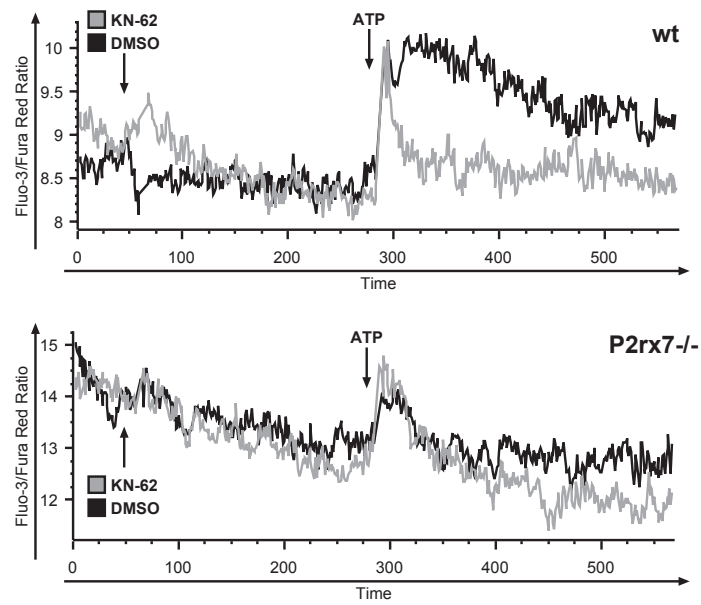

Supplement: Additional file 3: — P2X7-independent ATP signaling in BMMCs. A) wt and P2rx7-/- BMMCs were primed with 1 μg/ml LPS for 3.5 h and then left untreated or stimulated with 0.3 mM ATP or 0.3 mM ATPγS for the indicated times. TCL were then subjected to anti-P-Erk1/2 (upper panel) and anti-p85 (lower panel) immunoblotting. B) wt and P2rx7-/- BMMCs were stained with Ca2+-sensitive dyes Fura Red-AM and Fluo-3-AM (invitrogen, Darmstadt, Germany) in RPMI containing 12% FCS for 40 min at 37°C. Ca2+ flux was monitored by flow cytometry. Baseline fluorescence intensities of both dyes were set to the same height. After 30 sec vehicle or KN-62 was added for 5 min and than ATP [0.3 mM] was added for another 5 min. The ratio of Fluo-3/Fura Red * 10 was calculated and the results were converted to kinetics using FlowJo analysis software. [file s12964-014-0040-3-S3.pdf]
